# Medical History from the Earliest Times

**Published:** 1893-05-06

**Authors:** E. T. Withington


### Medical History from the Earliest Times.

By E. T. Withington, M.A., M.B. Oxon.

### LIII.—HARVEY, AND THE NEW PHYSIOLOGY.

"I dreamt I was in a vast catacomb, the air was thick and all was pitchy dark, the voice echoed, and swarms of bats were the only dwellers therein. And I thought, 'This is nothing else than the Tomb of Medical Truth,' and managed to make an opening which let in some light. Then me seemed that Galen tried to enter thereat with his small lamp; but he was fearful, and tottered, and fell full-length on the threshold, and upset his oil; yet when he rejoined his companions he told them wondrous tales of the cave, and they repeated all his stories, nor cared to investigate them. Next I saw Avicenna, and a crowd with him. He had learnt somewhat from Galen, yet got he not much further, for a giddiness came upon him, and he stumbled against a stone and fell prostrate; but when he got out he boasted none the less, in his strange tongue, that he had seen much more than his predecessors. Many followed, some in Galen's fashion, others in Avicenna's, but all fell; some ventured in with no light at all, and these saw nothing. At length I beheld Theophrastus Paracelsus enter. He had more foresight, and took a huge torch, and fastened a thread at the entrance that he might find his way back. So he penetrated further than any mortal before him, and went about the cave and saw many things. But the smoke from his torch filled the passages, and as he would have looked more closely at the Tomb of Truth, his strength failed and the light fell from his grasp. And lastly, I, poor mortal, ventured in with the dim light of a lantern at my girdle, and a thread hooked on behind that my hands might be free. And I saw much which those before me saw not; but being alone, I had not strength to accomplish what I would have done; and though I strove valiantly, the crowds of bats pressed upon me, so that I had to return with little profit."

So writes Van Helmont, though I have ventured to condense several pages into a paragraph. But while he was having this and other dreams, to be considered shortly, an English physician, one year his junior, was engaged in illuminating the Cave of Medical Truth, as with an electric light, whose beams pierced even the fogs from the smoky torch of Paracelsus. This was William Harvey (1578-1657), but we need not here describe what he discovered, or how he discovered it; may not the story be read by all in the shortest and most important of our medical classics, the immortal "Anatomical Exercise on the Motion of the Heart and Blood in Animals" (1628)? Our space will be better occupied by considering what was the precise effect and importance of the discovery, and why, in the history of our art, the name of Harvey is placed second only to that of Hippocrates.

Even the most vehement opponents of physiological research will probably admit that it is important for a physician to know that the blood circulates. But in the time of Harvey, and for more than a century afterwards, they might have argued that the discovery, and the numerous vivisections which led to it, had not only not produced any new and successful mode of treatment, but by giving rise to one-sided theories, such as those of the mechanical school, had rather retarded than furthered the progress of the healing art. The physiologists would doubtless have replied with Harvey that the new doctrine had let in upon them a flood of light and truth, had explained many problems and resolved many doubts, and had suggested the causes of many diseases and the best mode of treating them. They might have added that it had given its death-blow to the Galenic tyranny, had substituted observation and experiment for custom and authority, and had put a final end to such controversies as that between the Derivationists and Revulsionists, in which so much time and mental energy had been wasted. Could they have foreseen the future they might have finally pointed to the work of Harvey as the model for all later physiologists, and as the chief corner-stone of that sure foundation on which there should one day be built a rational and progressive science of medicine. "Before Harvey," says a distinguished French physician, "the sick man was looked upon from without—the symptoms; since Harvey, he is looked upon from within—the functions."

But we must admit that the new theory was at first a light-bringing rather than a fruit-bearing discovery; and the same has been the case with the countless lesser discoveries which have followed and resulted from that of the circulation. They may be compared to lamps set up in various parts of the above-mentioned cave, sometimes shedding light on things immediately useful to the practitioner, but more often revealing hidden passages, the exploration of which has resulted in increasing our knowledge of disease and its treatment in ways little dreamt of by the original investigators.

One such lamp had already been set in 1622 up by Gasper Aselli, Professor at Pavia, who, while demonstrating by vivisection the action of the recurrent nerves and diaphragm, noticed numerous white lines on the mesentery. Suddenly there flashed across his mind the tradition of peculiar mesenteric vessels seen by the ancient anatomists, and he cried out with Archimedes, "Heurēka, I have found it!" He had found the lacteals. But while Harvey left his discovery complete so far as was possible without the aid of the microscope, that of Aselli had to be finished and corrected by two medical students, Pecquet the Frenchman and Rudbeck the Swede. According to Galen the products of digestion are carried by the portal vein to the liver, and there converted into blood; and Aselli concluded, as a matter of course, that the lacteals also went to the liver. In 1647, however, Pecquet, a student at Montpellier, tired of "the dumb and cold science of anatomy," took to vivisection, and immediately discovered the receptacle of the chyle and the thoracic duct, which he traced to its termination in the veins. Three years later Rudbeck, a godson of the great Gustavus Adolphus, while studying medicine at Leyden, completed the investigation by finding the lymphatics, and he demonstrated these and the thoracic duct, which he had discovered independently of Pecquet, before Queen Christina of Sweden and a distinguished company in 1652. Both Pecquet and Rudbeck were great admirers of Harvey, whom they imitated even in the titles of the treatises, "New Anatomical Experiments," and "A New Anatomical Exercise," in which they announced their discoveries.

Finally, in 1661, four years after Harvey's death, ocular proof of the circulation was given by Malpighi of Bologna. He tells us that while examining the lung and mesentery of a frog with a lens, he noticed the contrary motion of the blood in the veins and arteries, but could not, at first, trace the connection between the two; till, on examining a piece of dried lung by transmitted light with a more powerful lens, he distinctly saw a network of fine vessels full of blood connecting the arteries with the veins. Aided by this, he beheld the wonderful spectacle of the circulation, first in the lung of a tortoise, and afterward in the lung and bladder of a frog; but it seems never to have occurred to him to examine the web of a frog's foot.

Thus, in a single generation, the whole aspect of physiology had been entirely altered, and the work was carried on with vigour for the rest of the century. We need only mention the Englishmen—Cowper, Glisson, Havers, Highmore, Lower, Willis, and Wharton; the Danes—Steno, Bartholinus, and Worm; the Germans—Kerkring, Meibom, Schneider, and Wirsung; the French—Vieussens and Duverney; the Swiss—Peyer and Brunner; the Dutch—De Graaf and Nuck; all contemporary physicians whose names are immortalised in various parts of the human body. The work of some of them will be referred to later on, but we must conclude for the present by noticing two discoveries, the great importance of which was long overlooked.

Dr. Richard Lower had proved, by experiments on animals, that the change from venous to arterial blood takes place only in the lungs, and only in the presence of atmospheric air; as, indeed, Servetus had asserted a century earlier. But his friend John Mayow (1645 79), one of the greatest physiologists of the seventeenth century, carried the investigation a step further, and showed that the agent of this change is a particular constituent of the air, which also supports combustion and is contained in nitre, and which he therefore called "nitro-aerial spirit." In short, he had discovered oxygen, though it had to be rediscovered a century later.

In September, 1683, Antony van Leeuwenhoek wrote from Delft to the secretary of the Royal Society announcing his discovery in the white matter between his teeth of microscopic animals "moving in the most delightful manner"; and he added a sketch in which we may clearly recognise the four chief forms of microbe, the longer and shorter rods of bacilli and bacteria, the minute spheres of the micrococci, and the corkscrew-like spirillum. He expresses his wonder that, in spite of his care in cleaning his teeth, his mouth should contain more "animals" than there are human beings in the united Netherlands, and says he had tried in vain to get rid of them by washing his teeth with vinegar. Sometime afterwards he began to take hot coffee, and the microbes disappeared, but they soon returned, inspite of continued coffee drinking. Leeuwenhoek says he hopes the Fellows of the Royal Society will be interested by this novelty, but he certainly had no idea that the discovery was destined one day to exert a greater influence both on medicine and surgery than even that of the circulation.

